# Enhanced warming and bacterial biomass production as key factors for coastal hypoxia in the southwestern Baltic Sea

**DOI:** 10.1038/s41598-024-80451-w

**Published:** 2024-11-27

**Authors:** Helmke Hepach, Judith Piontek, Hermann W. Bange, Theresa Barthelmeß, Anabel von Jackowski, Anja Engel

**Affiliations:** 1 Present Address: RD2, Biological Oceanography, GEOMAR Helmholtz Centre for Ocean Research Kiel, 24148 Kiel, Germany; 2 Biological Oceanography, Leibniz Institute for Baltic Sea Research Warnemünde, 18119 Rostock, Germany; 3 RD2, Chemical Oceanography, GEOMAR Helmholtz Centre for Ocean Research Kiel, 24148 Kiel, Germany; 4 Present Address: UMR7621 Laboratoire d’Océanographie Microbienne, CNRS/Sorbonne Université, 66650 Banyuls-sur-Mer, France

**Keywords:** Coastal hypoxia, Baltic sea, Bacterial biomass production, Time-series measurements, Stratification, Climate change, Long-term monitoring, Oxygen minimum events, Marine biology, Marine chemistry, Environmental impact, Carbon cycle

## Abstract

Coastal ecosystems are affected by a multitude of anthropogenic stressors. As the Baltic Sea ecosystems rank among the most altered marine ecosystems worldwide, they represent ideal model regions to study ecosystem responses to anthropogenic pressures. Our statistical analysis of data including dissolved organic carbon and nitrogen, as well as bacterial abundance and -biomass production from the time-series station Boknis Eck in the southwestern Baltic Sea reveals that bacterial biomass production intensifies towards summer following the phytoplankton spring bloom. Moreover, warming, especially very high temperatures in summer, enhances stratification and bacterial biomass production despite long-term reduction in nutrient input. A strong decrease in oxygen in the bottom layer is possibly linked to this. We detect an increasing trend in bacterial biomass production along with intensifying warming and stratification, and more frequently occurring hypoxia despite ongoing restoration efforts. If this trend continues, the coastal Baltic Sea ecosystem is likely to be altered even further. Coastal ecosystems play pivotal roles in mitigating impacts of climate change but if destroyed, they may amplify climate change further calling for stronger ecosystem management strategies.

**Supplementary Information:**

The online version contains supplementary material available at 10.1038/s41598-024-80451-w.

## Introduction

The global ocean is under constant pressure from anthropogenic activities resulting among other effects in strong warming, oxygen decline, and acidification. These changes have contributed to global shifts in the composition of marine communities, their biogeography, and consequentially to a reduction in ecosystem services^1^. Particularly climate change has had tremendous impacts on marine ecosystems. In a recent review, it was reported that 78.8% of studies focusing on climate change impacts on marine organisms found negative responses to warming^2^. This is especially true when warming interacts with other stressors such as eutrophication or deoxygenation. Hypoxia constitutes a severe threat to marine ecosystems and their services. It affects all aerobic organisms from a molecular to an ecosystem-wide level impacting global marine food webs^3^. Climate change has also been hypothesized to be one of the key drivers of the expansion of hypoxic zones, decreasing the solubility of oxygen, increasing stratification and potentially enhancing organic matter (OM) remineralization and the subsequential bacterial oxygen demand^4^.

Due to its unique hydrographical setting, environmental changes in the semi-enclosed Baltic Sea including warming and enhanced water column stratification^5^, acidification^6^, eutrophication^7^, pollution from contaminants such as pesticides^8^, and hypoxia^9^ have been observed in much higher rates than in other oceanic basins. Furthermore, climate change has led to an increased occurrence of marine heatwaves^10^, although it is under debate how they impact the marine environment (e.g. contrasting results in Humborg, et al.^11^ and Gindorf, et al.^12^). Several management strategies to restore the Baltic Sea ecosystem were enforced early on including HELCOM, the EU-wide Water Framework Directive (WFD) and the Marine Strategy Framework Directive (MSFD) coming into effect in 2000 (HELCOM and WFD) and 2008 (MSFD), respectively. It was therefore hypothesized that the Baltic Sea can be used as a “time machine” for estimating effects of anthropogenic pressures on the global ocean in the future and to assess the success of management strategies^13^. Time-series stations are particularly important tools for identifying and understanding ecosystem responses to climate-driven changes^14^. Long-term data from the Baltic Sea hence provide invaluable insight into effective protection measures for marine ecosystems on a global scale.

Coastal hypoxia in the Baltic Sea has significantly worsened on a long-term basis, both with respect to the number of sites experiencing hypoxic conditions and the frequency with which hypoxic events occur^15^. One reason for this is limited large-scale mixing of brackish Baltic Sea water with fresh, saline North Sea water. Large-scale transport of saline waters into the Baltic happens only through the Öresund, controlling deep water ventilation in the central basin^16^. Due to limited exchange with the North Sea and enhanced riverine input by over 250 rivers around the basin, the Baltic Sea is characterized by brackish water^17^. Ventilation by mixing oxygen from the surface layer into deeper layers is reduced by a strong pycnocline^18^. Warming further contributes strongly to hypoxia^19^.

The excessive external and internal inputs of nitrogen- and phosphorous-containing nutrients from river systems, shipping and atmospheric deposition remain a threat despite recent reduction efforts. About 22% less nitrogen and 18% less phosphorous were released to the southwestern Baltic Sea in 2020 compared to the reference period 1997 to 2003^20^. The release of inorganic phosphorous from anoxic sediments into the water column is an additional internal source. Resulting summer blooms, most prominently of diazotrophic cyanobacteria, further contribute to the OM load and hypoxic zones^21^.

The supply of dissolved organic matter (DOM; e.g. dissolved organic carbon (DOC) and dissolved organic nitrogen (DON)) is key in regulating the biological oxygen demand, as heterotrophic OM degradation is the major biological sink of oxygen^22^. The supply is largely driven by terrestrial sources, e.g. riverine input, in most of the coastal Baltic Sea regions. Terrestrial DOC (tDOC) makes up about 43% of total DOC^23^. The bioavailability of terrestrial DON (tDON) is potentially larger than that of tDOC, leading to a relatively steeper lateral gradient of DON from coastal areas to the Baltic proper^24^. The fraction of tDOM in the Baltic coastal regions most notably increases in spring and fall due to enhanced precipitation and riverine input. Simultaneously, autochthonous DOM rises in spring and fall due to phytoplankton blooms^25^.

Here, we explore the drivers of bacterial OM degradation and how this impacts bottom water oxygen depletion at the time-series station Boknis Eck (BE) between 2013 and 2019. BE is located in the southwestern Baltic Sea, and is one of the world’s longest-running marine time-series stations. Time-series data from BE include monthly measurements of inorganic nutrients, dissolved oxygen, temperature and salinity, as well as DOC and DON, bacterial abundance and bacterial biomass production (BBP). We further compare our data with previous measurements of bacterial abundance and BBP measurements at the same location from 1991 to 2008^26^. Due to differences in methods used to measure both bacterial abundance and BBP in the time periods of 1991 to 2008 and 2013 to 2019, we only take these additional data into account when comparing long-term trends. Previous analysis of inorganic nutrient and oxygen data from BE revealed a constant decline of dissolved oxygen in the bottom water. This study aims at investigating the hypothesis that microbial activity in concert with rising temperatures substantially contributes to the continued decline in oxygen despite improvement efforts such as the substantial reduction of inorganic nutrient inputs^27^.

## Results and discussion

### Physical parameters and oxygen

**Fig. 1:**
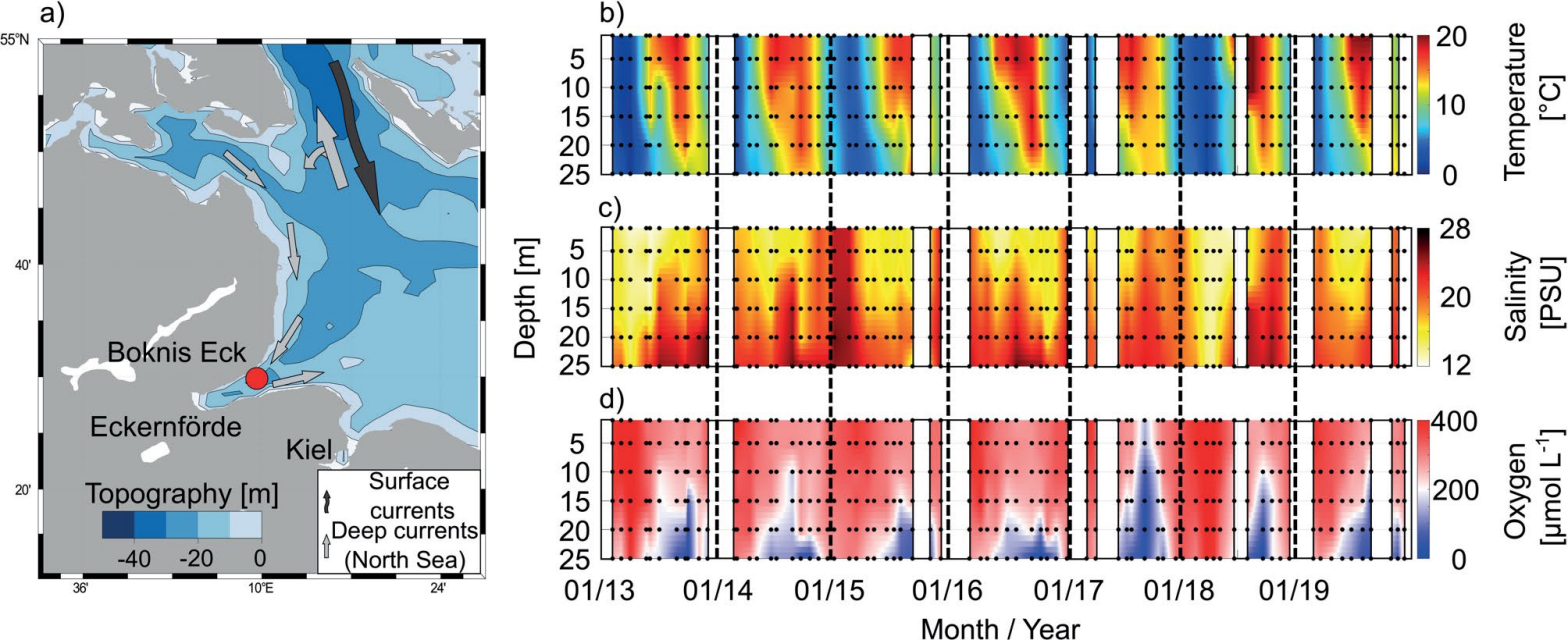
Boknis Eck location in (**a**), temperature, salinity, and oxygen in (**b**–**d**), respectively. Dots in (**b**–**d**) indicate sampling times and depths; white gaps mean that there was no sampling in this month. See Fig. S1 in the supplement for the overarching region. Data are shown in the time frame of 2013 to 2019. The map in (**a**) was generated using the M_Map toolbox in Matlab^®^ 2023a^29^.

Temperature and salinity (Fig. 1, Table S1) between 2013 and 2019 show clear seasonal cycles. The lowest water temperatures predominantly occurred in March. Cold temperatures in 2015 were accompanied by the highest salinities, when more saline North Sea water is transported into the Baltic Sea. Water temperatures started to increase in the upper water column in spring leading to an onset of enhanced stratification (temperature gradient ∇T ≥ 0.6 °C m^− 1^)^27^. Stratification usually persisted from May or June to sometime between September and November with the upper 10–20 m typically as the mixed layer (Table S2). Water temperatures steadily increased during the year especially in the upper water column, usually reaching the annual surface water maximum in August or September, and the bottom layer temperature maximum in September or October (Table S1; Fig. 1). Temperatures indicating category I marine heatwaves in the bottom water according to Hobday et al.^28^ could be identified in four out of seven years (Fig. S2; 2014, 2015, 2017, 2019).

Oxygen concentrations showed a pronounced seasonal cycle at BE (Fig. 1d). Highest oxygen concentrations were observed in winter and spring in the well mixed water column (Table S1). Concentrations steadily decreased in the lower stratified water column towards summer, usually reaching minimum concentrations in September with 178 ± 110 µmol L^− 1^ (all September data 1–25 m). Very low concentrations to below the detection limit (~ 2 µmol L^− 1^) were observed in the bottom layer at 25 m in 2014 (September to October) and in 2016 (September to November). Especially the year 2014 was characterized by heatwave-like temperatures throughout the year (Fig. S2).

### Biogeochemical parameters

**Fig. 2:**
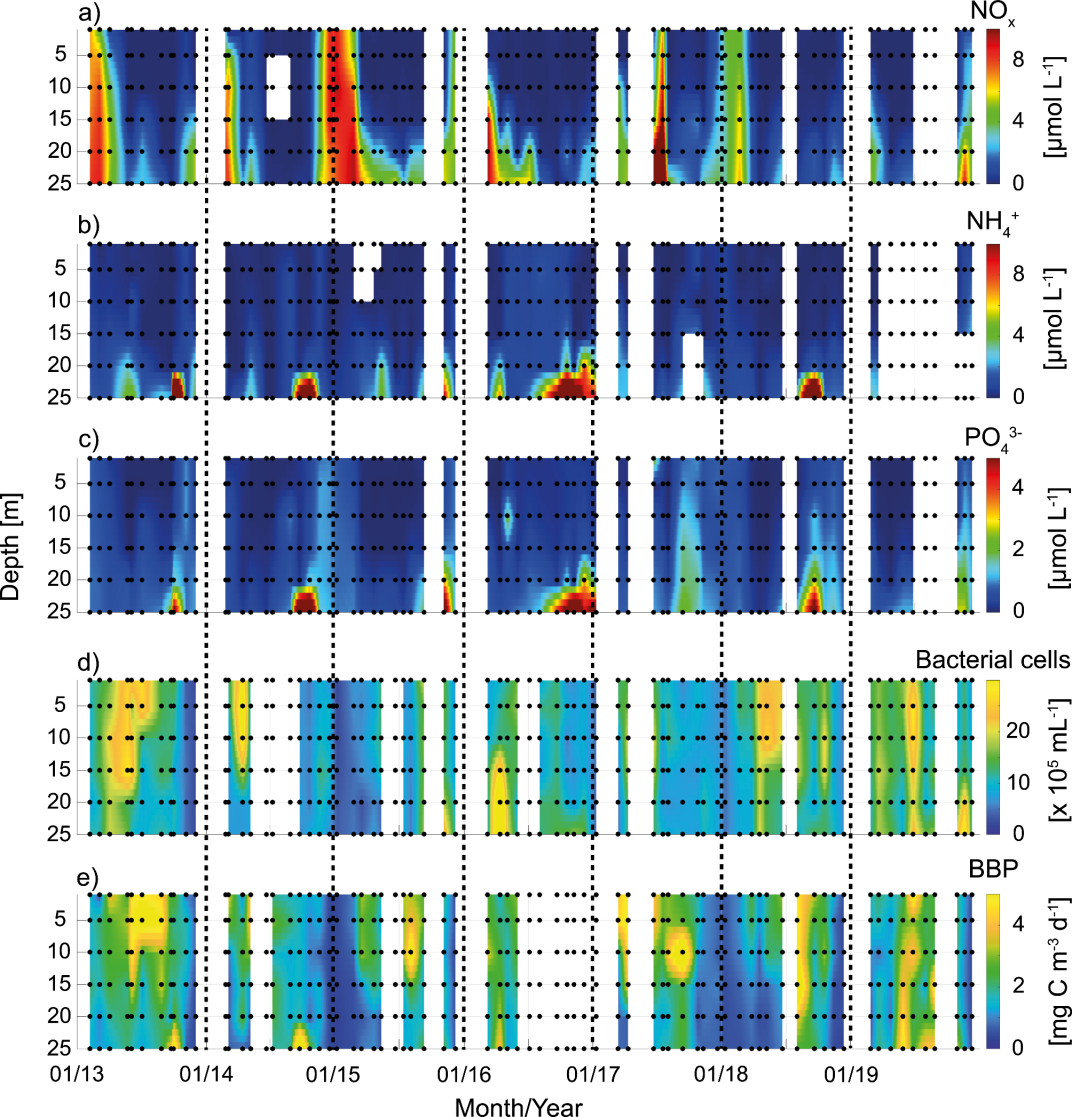
Time series of depth profiles of NO_x_ (NO_2_^-^ + NO_3_^-^) in (**a**), NH_4_^+^ in (**b**), PO_4_^3-^ in (**c**), bacterial abundance in (**d**), and bacterial biomass production (BBP) in (**e**). Dots implicate sampling times and depths; white gaps mean that there was no sampling in this month. Data are shown in the time frame of 2013 to 2019.

NO_x_-concentrations (the sum of nitrate (NO_3_^−^) and nitrite (NO_2_^−^); Fig. 2, Table S1) were overall enhanced in fall and winter, decreasing strongly in spring and summer especially in the upper water column due to the blooming season. Pre-bloom concentrations ranged from 2.1 ± 1.1 µmol L^− 1^ (2017, *n* = 12) to 8.0 ± 1.7 µmol L^− 1^ (2015, *n* = 12). Ammonium (NH_4_^+^) concentrations were low in winter and summer, and peak NH_4_^+^ was usually observed during fall in the bottom layer coinciding with the lowest oxygen concentrations. NH_4_^+^ likely originated from dissimilatory NO_3_^−^ reduction processes in the sediment^21,30^. Phosphate (PO_4_^3−^) displayed similar seasonal patterns. Concentrations of PO_4_^3−^ were slightly elevated in the early winter months declining in spring. Pre-spring bloom concentrations ranged from 0.5 ± 0.2 µM (2014, *n* = 12) to 1.5 ± 2.7 µM PO_4_^3−^ (2017, *n* = 6). Highest PO_4_^3−^ concentrations coincided with low oxygen in the bottom water between September and November with the maximum concentration (11.3 µM) in November 2016. The remobilization of PO_4_^3−^ from anoxic sediments was suggested to be one of the main drivers of eutrophication in the Baltic Sea^31^. The described seasonal dynamics of inorganic nutrients agree with long-term measurements at several Baltic Sea stations^21^.

The average ratio of DIN (dissolved inorganic nitrogen from NO_x_ and NH_4_^+^) to DIP (dissolved inorganic phosphorous from PO_4_^3−^) was 6.9:1. The observed dynamics of DIN: DIP are consistent with previous observations: shifting ratios from phosphorous- (winter and spring) to nitrogen limitation (summer and fall) due to input of DIN in winter time, following drawdown of DIN after the spring bloom, and release of PO_4_^3−^ in summer from the sediment^21,32^. Declining anthropogenic NO_3_^−^ and rising internal DIP due to more frequently occurring hypoxia shift the coastal Baltic Sea largely towards nitrogen limitation^31,32^.

Bacterial cell abundance (Fig. 2d, Table S1) between 2013 and 2019 was often characterized by two maxima in the course of the year: typically, a stronger maximum was observed between March and June, and a second, usually less pronounced maximum could be detected between September and December. BBP (Fig. 2e) showed the most pronounced maxima between August and October.

DOC (Fig. S3, Table S1; measured from 2016 on) maxima were mostly observed in spring and early summer. Concentrations and the observed seasonal variability were in range of other coastal stations in the Baltic Sea (290 ± 25 µmol L^− 1^ at Heiligendamm^33^; 360 µmol L^− 1^ in the Vistula Estuary^34^). TDN and DON were high in winter likely due to enhanced precipitation and associated land runoff, decreased towards March potentially due to DON utilization by phytoplankton in the spring bloom (e.g. Moschonas et al., 2017^35^), depleting further towards late summer simultaneously with enhanced BBP. The observed concentration range of DON is in agreement with other coastal Baltic Sea stations (17.2 ± 1.6 µmol L^− 1^ at Heiligendamm^33^; 16 µmol L^− 1^ at Vistula Estuary^34^).

### Origin of dissolved organic carbon and nitrogen in the water column

**Fig. 3:**
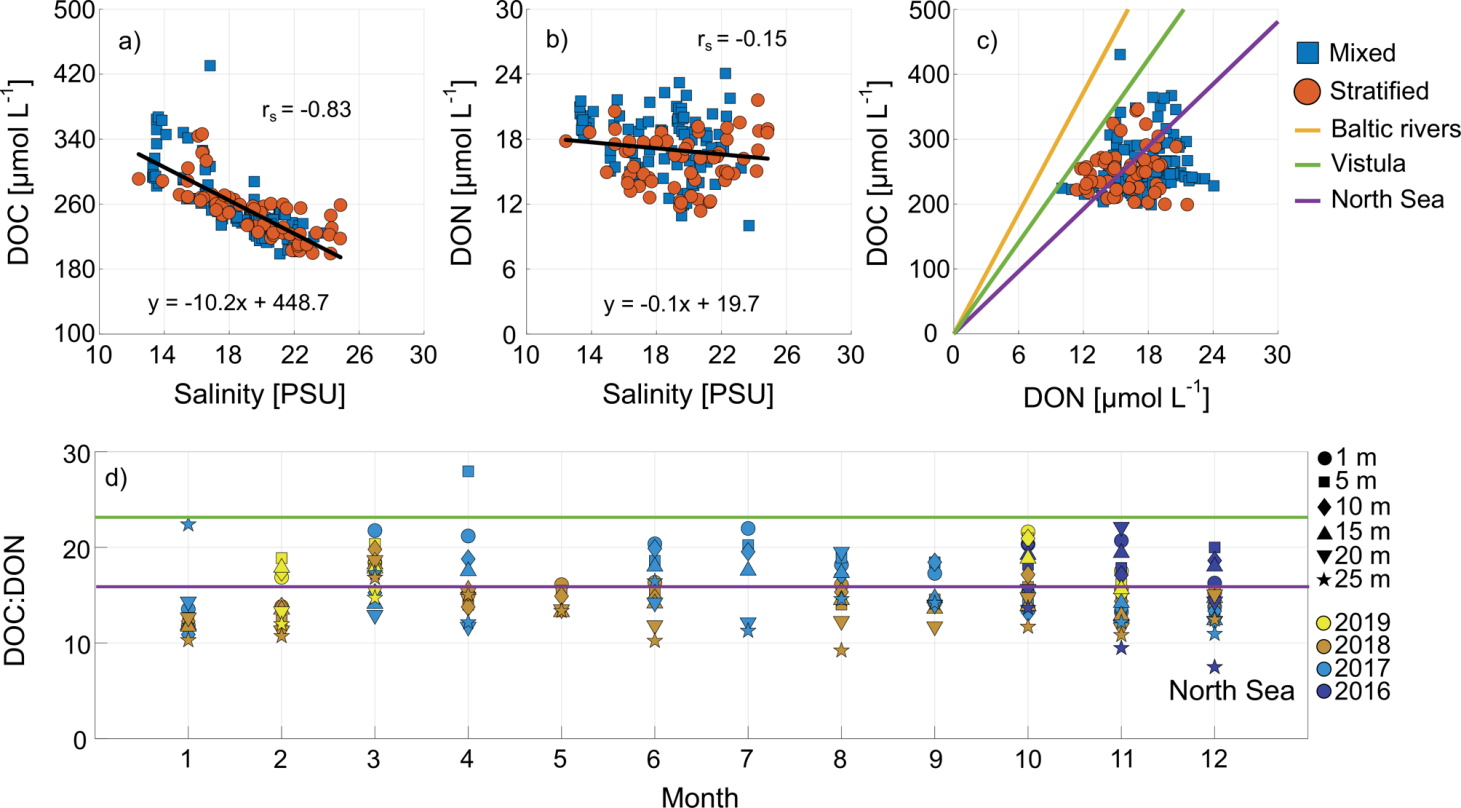
DOC and DON vs. salinity in (**a**) and (**b**) with regression lines, and DOC vs. DON in (**c**) with the ratio found in Baltic rivers^36^, the Vistula Estuary^34^, and the southwestern North Sea in winter time^37^. The ratio of DOC and DON is shown in (**d**) along with the lines indicating the average ratios from the Vistula Estuary and the North Sea (no ratios were higher than the Baltic river ratio). The symbols in (**a**) to (**c**) refer to the mixed and stratified seasons. Data are shown in the time frame of 2016 to 2019.

The quality and quantity of OM contributes significantly to determining the magnitude of bacterial carbon uptake^38^. DOC was significantly inversely correlated with salinity (r_s_ = -0.83; Fig. 3a). Saline water was associated with lower DOC concentrations, likely resulting from the influx of water from the North Sea containing less tDOC than coastal Baltic Sea water^33,37^. Low salinity water with higher tDOC concentrations potentially originates from submarine groundwater discharge and land run-off due to precipitation^39^. Deviations from this relationship above the correlation line may hint to autochthonous sources of DOC especially during bloom seasons^24^ (Fig. S4 in the supplement shows the potential input of autochthonous DOC per month). The correlation of DON with salinity was not significant (Fig. 3b), indicating that DON dynamics are not primarily driven by physical mixing processes. The significant relationship of DOC and salinity and the non-significant relationship of DON and salinity apply to both the mixed and the stratified seasons.

The ratio of DOC and DON can provide further information on the origin of DOM. In a similar approach as in Voss et al.^34^, DOC: DON was compared to regression lines from a range of Baltic Sea rivers (ratio: 31.0; terrestrial sources^36^), the Vistula Estuary (ratio: 23.5; mixture of terrestrial and marine DOM^34^), and the southwestern North Sea in winter time (ratio: 16.0; mostly marine DOM^37^; Fig. 3c). Ratios lying between the Baltic Sea river- and the Vistula Estuary lines can be assumed to refer to DOM of mainly terrestrial origin, ratios between the Vistula- and North Sea line potentially originate from mixed autochthonous and allochthonous sources, while ratios below the North Sea line show characteristics of mainly marine DOM. All of the DOC: DON ratios were below the ratios of Baltic rivers indicating that DOM at BE originated from mixed (above the North Sea line) and mainly autochthonous sources (below the North Sea line).

The slope of the model type II regression line of DOC vs. DON has previously been used to determine the potential ratio of DOM production and consumption both by autotrophic and heterotrophic processes assuming a similar ratio for both^40^. This approach was used to determine potential production- and consumption ratios at BE for the ratios between the Vistula and North Sea lines (mixed origin), and for the data below the North Sea line (mainly marine DOM; calculated type II regression lines in the supplement in Fig. S5). DOC of mixed origin was significantly correlated to DON (r_s_ = 0.88, *p* ≤ 0.05; Fig. S5) with a slope of 18.0, implying slow remineralization and large carbon production in comparison to nitrogen as can be observed during the exponential phytoplankton bloom phase (mostly observed in March at BE)^40,41^, or large allochthonous input of carbon-rich DOM. DOC and DON below the North Sea line (mostly autochthonous origin) correlated significantly with each other but with a lower r_s_ of 0.41 (*p* ≤ 0.05) and a slope of 10.3 implying stronger remineralization of the respective DOM^40^ during late summer and early fall as most ratios measured during this time frame lay in this cluster (Fig. 3c,d). The decrease of ratios indicating more labile and autochthonous DOM towards summer is especially visible in the lower water column. This overlaps with higher BBP during the same time and at the same depths.

### Relationships of bacterial biomass production and environmental parameters

Principal component analysis was performed on the whole dataset after data normalization including BBP and bacterial abundance, temperature, salinity, oxygen and nutrient data (Fig. S6) as a first point of reference for further analysis (DOM data was not included due to limited data availability). While component 1 (PC1) was mainly driven by salinity, temperature was the main driver of component 2 (PC2). Since stratification has been indicated to play a significant role in determining oxygen levels at BE^27^, the data set was divided into the mixed and the stratified season (Table S2) to identify whether drivers of BBP differed between the seasons. We found no significant differences with respect to salinity (PC1) between the two seasons. Means and the variance of oxygen (PC1), temperature (PC2), and BBP (PC2) from the mixed season were all significantly different from the stratified season implying different drivers within each season (Fig. S7). The correlation of PC2 with BBP was significant, indicating similar patterns in temperature and BBP.

**Fig. 4:**
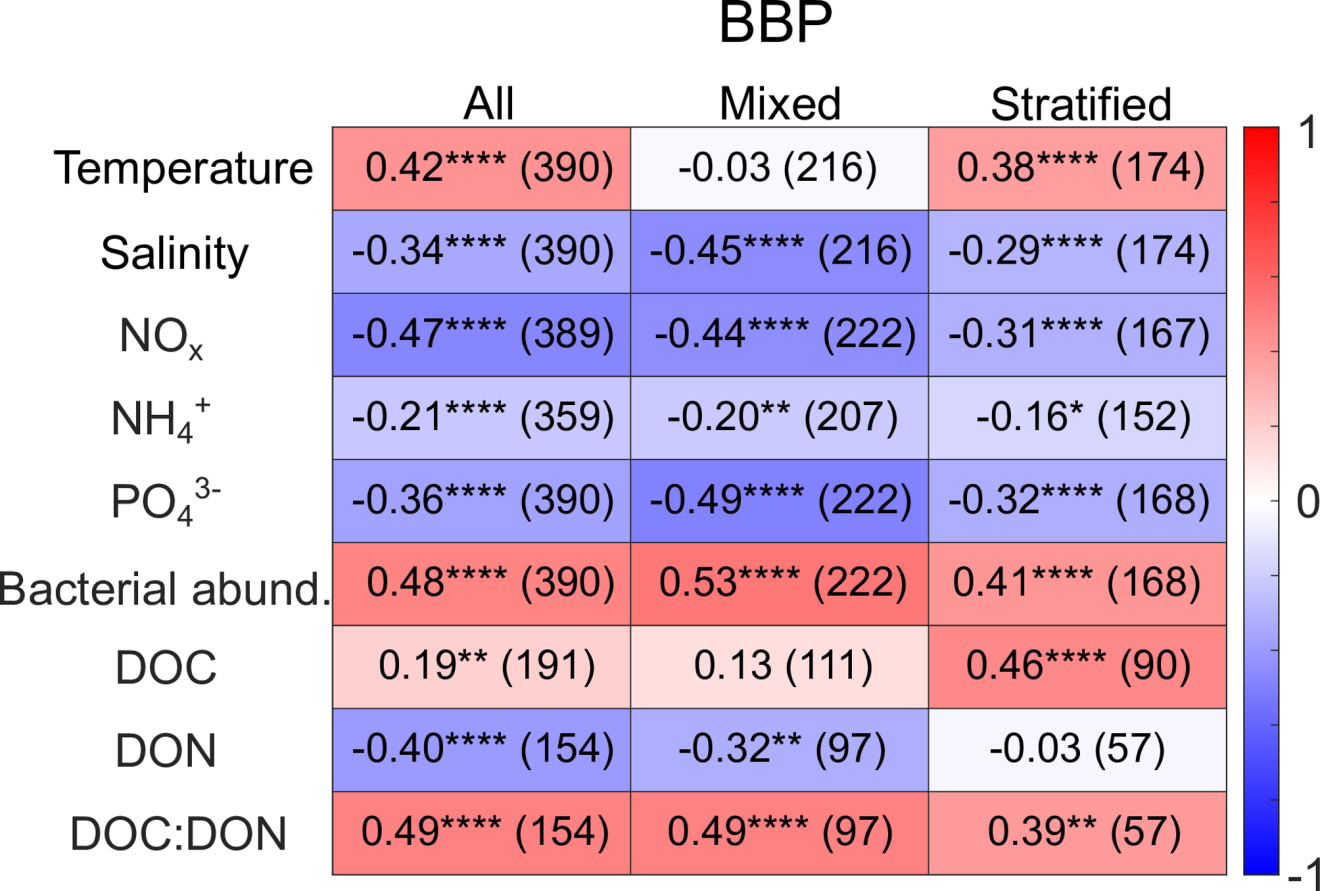
Spearman’s rank correlation coefficients (r_s_) listed on a heatmap for BBP and all environmental parameters except for oxygen. The first column includes all data from 2013 to 2019, which were further separated into data from the mixed and the stratified season (Table S2). Asterisks indicate significance levels; no asterisks indicate *p* > 0.05. The number of data points in each correlation are listed in the brackets.

The PCA showed that patterns in the parameters differ with respect to the respective season, which was further explored in a correlation analysis (all datapoints, mixed season, stratified season; Fig. 4). Please note, the correlation analysis performed in this manuscript can only provide hints for causalities, which in future studies should be confirmed by laboratory-based incubation studies.

As indicated by the PCA, temperature correlated positively with BBP across data from both seasons together and in the stratified season. The most significant correlation with respect to salinity and BBP was found in the mixed season (inversely correlated; Fig. 4), indicating that the most active bacterial communities in the mixed season were associated with lower salinities in the top layer where also phytoplankton blooms occur. Bacterial abundance correlated with BBP in all seasons, and was the most significant contributor to BBP variability in the mixed season (Fig. 2). The less pronounced relationship of bacterial abundance and BBP in the stratified season implies that environmental factors and substrate availability from e.g. DOM largely impact the magnitude of BBP in this season. Additionally, viral lysis and grazing by protozoa may contribute to the decoupling of bacterial abundance and BBP. Previous studies have found that protistan grazing may enhance metabolic activity while simultaneously impacting seasonal succession patterns in bacterial biomass^42,43^.

NO_x_ and PO_4_^3−^ correlated inversely with BBP taking into account all data, the mixed season and the stratified season. The observed nutrient depletion in late spring and fall at BE is likely a result of phytoplankton blooms^26^ along with further uptake of inorganic nutrients by the bacterial community. This is supported by a weak positive, but significant relationship of BBP with DIN: DIP in the mixed season (r_s_ = 0.19*, not shown). BBP has been shown previously to intensify in succession of phytoplankton blooms in the Baltic Sea^44,45^.

BBP correlated with DOC: DON with respect to the whole data set, the data from the mixed season, and less significantly in the stratified season (all positive correlations). The availability of DOC and DON for uptake by the bacterial community is strongly dependent on the sources of DOM in the Baltic Sea. While tDOC is less bioavailable^24^, tDON may be more accessible for the heterotrophic community^46^, which supports a negative relationship of BBP and DON. For the interpretation of the positive significant correlation of BBP with DOC in the stratified season, it needs to be noted that the stratified season between 2016 and 2019 included the month October (Table S2). The onset of the fall phytoplankton bloom in the western Baltic Sea can often be observed between September and October^47^, leading to production of autochthonous DOC, and thus a positive significant correlation in the stratified season (see Fig. S4 of the theoretical input of autochthonous DOC). Lower DON due to heterotrophic uptake and higher autochthonous DOC during the spring and fall blooms may explain a positive correlation of BBP with DOC: DON (higher DOC compared to DON).

During the stratified (summer) season, the correlations of BBP with temperature and DOC tentatively confirm previous assumptions that temperature plays a pivotal role in regulating the flow of carbon into the heterotrophic community in the Baltic Sea. Results from studies focusing both on short-term warming experiments^48^ and field data^49,50^ in the Baltic Sea show a decoupling of early phytoplankton bloom stages and active bacterial growth suggesting that seasonal temperature increase is needed to stimulate bacterial responses to autotrophic carbon production.

### Impact of temperature on bacterial production and oxygen

Uncertainties regarding the interactions between OM remineralization and oxygen consumption remain^51^. This is mainly due to methodological limitations of measuring microbial respiration in the field leading to a low amount of available data^52^. This uncertainty hinders their representation in biogeochemical models and in future oxygen projections^53^ especially with regard to the magnitude of its depletion and spatial patterns^54^. Parallel measurements of bacterial remineralization and BBP are rare (e.g. Vikström and Wikner^55^, García-Martín et al.^56^, González-Benítez et al.^57^). The bacterial growth efficiency, representing the equilibrium between bacterial growth and energetic demand, in the Baltic Sea has been found to average around 0.24^58,59^ especially during late spring and summer. Similar seasonal patterns of BBP and bacterial respiration with maxima in summer were observed at a time-series station in the Baltic proper^59^.

**Fig. 5:**
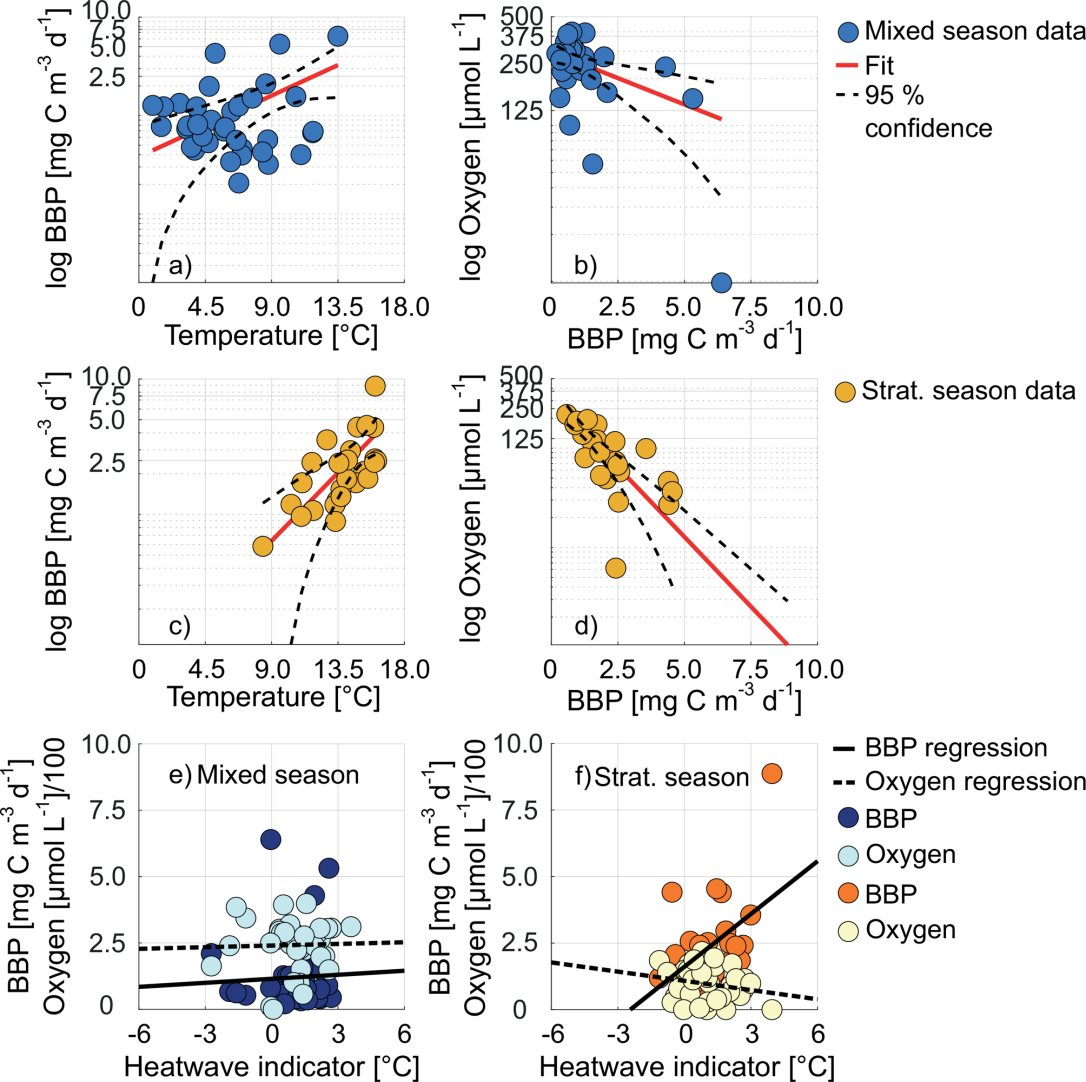
BBP from 25 m vs. the mean temperature in the water column for the mixed season are shown in (**a**). Oxygen is plotted vs. BBP for the same time frame in 25 m in (**b**). BBP vs. mean temperature (25 m) and oxygen vs. BBP (25 m) within the stratified season are shown in (**c**) and (**d**). Dots refer to data points. Red lines represent the fit of the datasets. Dashed lines indicate the 95% confidence intervals of the fits. Fit parameters are listed in the supplement (Table S3). BBP and oxygen vs. the heatwave indicator are depicted in (**e**) in the mixed season and in (**f**) in the stratified season. Data are shown in the time frame of 2013 to 2019.

Previous analysis of long-term trends of temperature and oxygen at BE revealed that temperatures increased strongly in the surface layer, and oxygen decreased significantly in the bottom layer since 1957^27^ (about 0.73 µmol L^− 1^ yr^− 1^ of oxygen lost until 2019). The strong oxygen decline despite reductions in the nutrient budget was hypothesized to be the result of temperature impacting both the stratification of the water column and remineralization rates^27^. However, this hypothesis could not be tested in the mentioned study as bacterial parameters were not included. We tested this hypothesis now using our BBP measurements between 2013 and 2019 by dividing the data set for 25 m into the stratified and the mixed season, and plotted BBP vs. temperature and oxygen vs. BBP, each for the stratified season and for the mixed season (Fig. 5). The coefficients of determination for the fits were r^2^ = 0.36 for BBP and temperature (significant relationship between modeled and measured data, Table S3), and r^2^ = 0.70 for oxygen and BBP (not shown: the r^2^ for temperature and oxygen was 0.63) for the stratified season (significant relationship between modeled and measured data, Table S3). Less variance could be explained for the same parameters in the mixed season: the coefficients of determination were r^2^ = 0.13 for BBP and temperature, r^2^ = 0.23 for BBP and oxygen (no significant relationships between modeled and measured data, Table S3). The non-significant relationships of temperature, BBP and oxygen in the mixed season and the significant relationships in the stratified season implied that BBP influences oxygen mainly during the stratified season. The large part of oxygen variability explained using BBP as predictor in the stratified season tentatively confirms the previously proposed hypothesis^27^: temperature affects water column stratification, which is prolonging due to ongoing climate warming, and bacterial activity. The prolonged time, in which bacterial activity may draw down oxygen in the lower water column, which cannot be replenished with oxygen from the top layer, increases the likeliness of the bottom layer to become anoxic. Temperature as driver of BBP is in agreement with previous studies^49,60^, in which warming led to enhanced respiration and elevated bacterial biomass especially under eutrophic conditions in the Baltic Sea. Our analysis suggests that although nutrient inputs have strongly decreased, the prolonged and intensified stratification season along with enhanced bacterial activity has led to a more frequent occurrence of oxygen minimum events at BE, threatening ecosystem integrity. The effect of changing solubility due to temperature is discussed in the following section.

Furthermore, the potential impact of heatwaves on BBP and oxygen was investigated using a heatwave indicator (Fig. 5e for the mixed season and 5f for the stratified season). The heatwave indicator was calculated based on the time-series from 1957 until the respective year with values below zero indicating deviations below the long-term mean and above zero above the long-term mean. The relationships between the BBP and oxygen data and the heatwave indicator were not significant (*p* ≤ 0.05) neither in the mixed season (r_s_ = 0.02, *p* = 0.92 for BBP, r_s_ = 0.00, *p* = 0.98 for oxygen) nor in the stratified season (r_s_ = 0.30, *p* = 0.30 for BBP, r_s_ = −0.16, *p* = 0.36 for oxygen). Yet, a tendency of particularly warm summers fostering more active oxygen drawdown can be observed. This indicates that a potential rise in the occurrence of heatwaves may further enhance the observed temperature effect on the formation of seasonal coastal oxygen minimum zones in the future, which should be investigated in future studies.

Assuming that the trend of warmer summers, as well as increasing internal nutrient loadings and bacterial activity continues, the occurrence of hypoxic events may increase even further in the future counteracting ongoing restoration efforts.

### Long-term trends

Investigating long-term trends provides useful information on biogeochemical dynamics. Please note, different methods were used for bacterial abundance (epifluorescence microscopy) and BBP (thymidine incorporation) from 1991 to 2008^26^. Epifluorescence microscopy and flow cytometry has previously been shown to produce similar results in aquatic ecosystems^61,62^. Thymidine incorporation was earlier found to be comparable to leucine incorporation but may potentially lead to slightly larger estimates (slope: 0.76–0.92^63^).

**Fig. 6:**
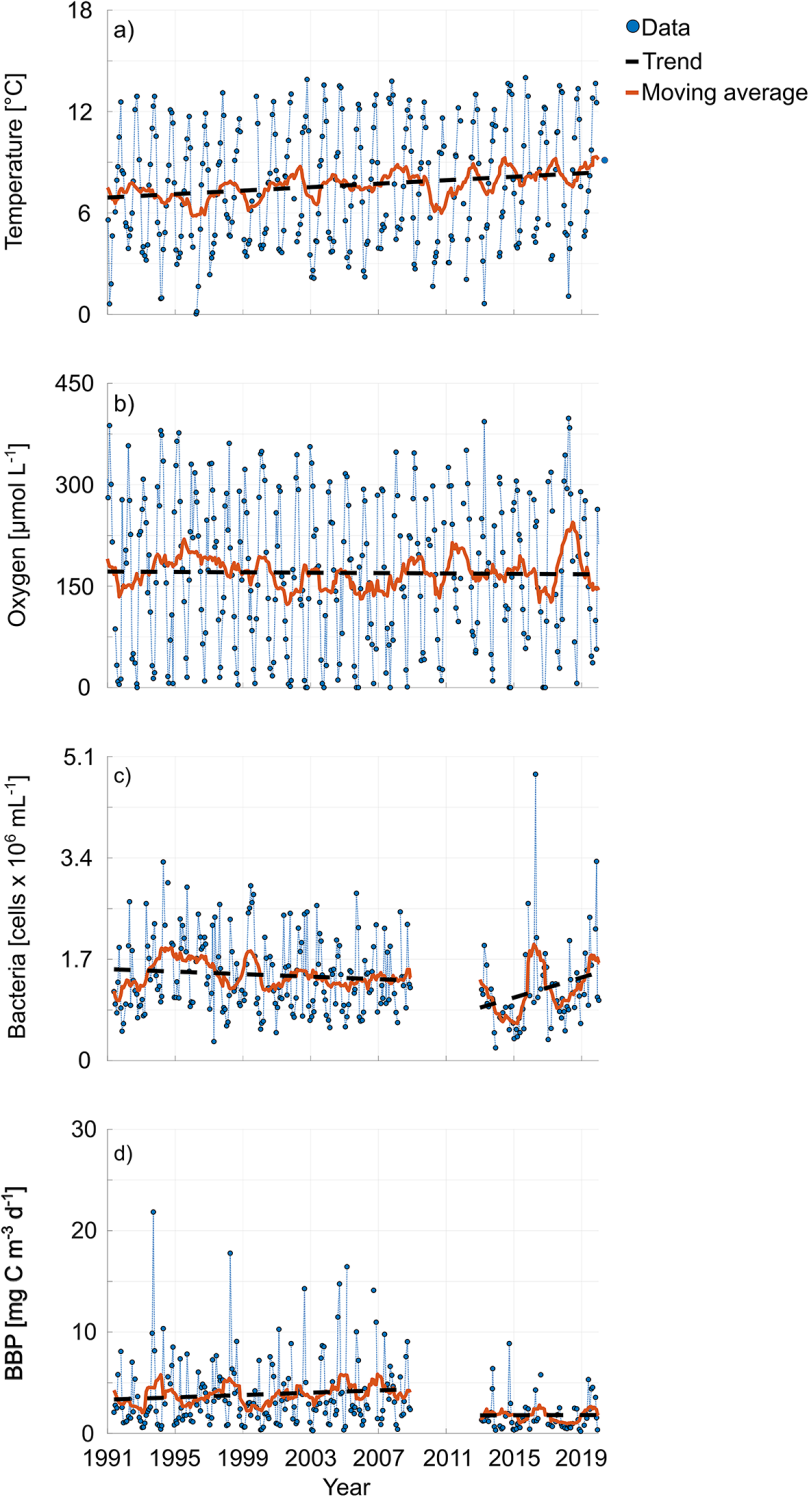
Long-term trends in temperature (**a**), oxygen (**b**), bacterial cell abundance (**c**) and BBP (**d**), each from 25 m, were calculated from the 12-months moving averages using linear regression. Data are shown in the time frame of 1991 to 2019.

Long-term data including data from Hoppe et al.^26^ and from this study were evaluated with respect to all available months (Fig. 6a–d). Trends were calculated separately for bacteria and BBP due to the data gap between 2008 and 2013. All long-term trends with respect to all months were tested for significance using the Mann-Kendall Test. All trends were calculated to be significant except for BBP between 2013 and 2019 (Table S4). Furthermore, the Mann-Kendall Test was applied to data only in August and September when BBP in the lower water column usually reached its maximum and oxygen its minimum.

The trend of increasing water temperatures and declining oxygen, both with respect to the whole year and considering only the stratified season, has continued since the last assessment^27^ (Fig. 6; Table S4). This becomes especially apparent when comparing the two time periods of 1991 to 2008 and 2013 to 2019. Both the temperature increase and the oxygen decrease have intensified in the more recent time period. Considering only the data from August and September, a significant negative trend in oxygen was observed between 2013 and 2019, confirming a more frequent occurrence of seasonal oxygen minimum events in late summer and early fall in recent years. Additionally, we observe an enhancement in thermal stratification since 1991 both with respect to the strength of stratification (slope: 0.07 °C m^− 1^ yr^− 1^, Fig. S8 in the supplement) and the length of the stratified season within one year (from about 3 months to 5 months, Fig. S8 in the supplement). Despite reductions of the nutrient input, hypoxic conditions are now becoming an almost yearly feature in the coastal southwestern Baltic Sea.

While bacterial abundance showed a negative trend until 2008, this has reversed since 2013. The positive trend in bacterial biomass in the last decade is mainly driven by bacterial biomass in spring and early summer. This may be attributed to stabilizing nutrient inputs, increasing temperatures^60^, and a subsequential earlier onset of the spring phytoplankton bloom^64^. No significant trend was identified for bacterial abundance in August neither between 1991 and 2008, nor between 2013 and 2019. BBP was observed to rise in both time periods, however, the trend was not significant between 2013 and 2019 (Table S4). Considering only August and September, which are the pivotal months with respect to oxygen drawdown, we see a positive significant trend in BBP in the time period 2013 to 2019, which goes along the negative trend in oxygen in the same time period. To determine the physical effect of temperature increase on oxygen solubility, the theoretical decrease in solubility was calculated according to Garcia and Gordon, 1992^65^ for the rates of change in temperature between 1991 and 2008 and between 2013 and 2019 (August and September). While solubility changes may account for about 71% of the change in bottom layer oxygen between 1991 and 2008, a lesser amount, 54%, of the change in oxygen concentrations may be explained by temperature-driven solubility changes between 2013 and 2019. This implies that the impact of biological processes on bottom layer oxygen concentration has risen in the last years. Oxygen decrease is likely a result of strong warming and consequential stratification, which enhances oxygen depletion in the bottom layer on a long-term basis.

## Summary and implications 

In this study, we investigated long-term trends of several environmental parameters such as oxygen, bacterial abundance and – biomass production (BBP) in the southwestern Baltic Sea at the time-series station Boknis Eck with special emphasis on the impact of warming. BBP was shown to enhance after the occurrence of the spring bloom with maximum BBP rates in summer supported by inorganic nutrient and DOM data based on a comprehensive correlation analysis. Temperature was shown to impact summer oxygen decline in the bottom layer of the southwestern Baltic Sea, which is potentially influenced by two mechanisms: (1) the stratification season is now prolonging with continued warming, and (2) BBP in the stratified season has intensified. Although efforts to reduce inorganic nutrient loadings to the coastal Baltic Sea have been successful (reduction of inorganic phosphorous and nitrogen influxes by 18–22%^20^), our study reveals that induced warming has an opposite effect on these efforts, preventing the coastal Baltic Sea ecosystem from recovering. The supply of inorganic and organic nutrients is seemingly still high enough to support elevated BBP in summer. Assuming that warming will continue to worsen along with a more frequent occurrence of heatwaves, the coastal Baltic Sea ecosystems will continue to degrade with severe impacts on their services. With regard to ecosystem management, large-scale monitoring of the bioavailability of OM present in the water column along with bacterial biomass abundance and BBP should thus be included in future restoring efforts. In light of our field data, strong efforts to reduce eutrophication including organic nutrients need to continue, since the impact of temperature on bacterial OM degradation is potentially less severe under oligotrophic conditions. This together with impacts on metabolic rates due to temperature need to be investigated in process-oriented laboratory studies to decrease the uncertainties in the knowledge of these processes, and to take effective countermeasures.

## Methods

### Sampling site

The BE time-series station in the Eckernförde Bay is located at 54°31’ N and 10°02’ E, and has a water depth of 28 m. Sampling of physicochemical parameters started in 1957. Bacterial abundance and BBP were recorded from 1988 to 2008^26^, and again from 2013 until 2019. The Eckernförde Bay is impacted by inflow events of saline water from the North Sea, with a dominant flow into the bay from the North (Fig. 1). Stratification of the water column is induced by increasing temperatures in spring and can last until early fall. Phytoplankton blooms usually occur in spring and fall^66^. While local riverine input of inorganic nutrients and tDOM is negligible, direct runoff from land needs to be taken into consideration^27^. The station is monthly sampled at six standard depths (1, 5, 10, 15, 20, and 25 m) using Niskin bottles.

### Hydrographical data, oxygen and inorganic nutrients

Temperature and salinity were measured with a CTD attached to a rosette (Hydro-Bios, MWS 6). Samples for dissolved oxygen were taken onboard in Winkler bottles, fixed, shaken for at least 30 s, and analyzed within several hours on the same day using Winkler titration^67^.Triplicate samples for determination of inorganic nutrients using Segmented Continuous Flow Analysis were stored at −20 °C until analysis^67^. Measured inorganic nutrients include NO_x_ (nitrate (NO_3_^-^) + nitrite (NO_2_^-^)), ammonium (NH_4_^+^) and phosphate (PO_4_^3-^).

### Dissolved organic carbon and nitrogen

For the analysis of DOC and total dissolved nitrogen (TDN) between October 2016 and December 2019, 20 mL of seawater were filtered through 0.45 μm GD/X-filters (Whatmann, UK) into combusted glass ampules (8 h, 450 °C). Duplicate samples were acidified with 20 µL of 30% ultrapure hydrochloric acid, sealed, stored at + 4 °C, and analysed by a high-temperature catalytic oxidation method using a TOC analyser (TOC-VCSH) from Shimadzu^68^. DOC and TDN measurements are available from October 2016 on. DON was derived by subtracting NO_x_-N and NH_4_^+^-N from TDN.

### Bacterial cell numbers and bacterial biomass production

Samples for bacterial cell counts and BBP were taken in duplicates. A volume of 1.7 mL of seawater was fixed with 85 µL of glutardialdehyde (GDA, 25%), gently mixed, incubated at room temperature for 15 min, and stored at −80 °C until analysis. Bacterial cells were stained with SybrGreen I (Thermo Fisher Scientific, USA), counted using a flow cytometer (FACSCalibur, Becton Dickinson, USA) with a detection limit of 2000 events s^− 1^, and calibrated with TruCount beads (Becton Dickinson). Cell numbers were estimated after visual inspection and manual gating in the cytogram of side scatter versus green fluorescence, using the software CellQuest Pro (Becton Dickinson). Yellow-green latex beads (1 μm, Polysciences) served as the internal standard.

BBP was measured by^3^H-Leucine incorporation under oxic conditions. 1.5 mL of sample from each depth together with one poisoned control per sampling day were incubated at in situ surface temperature for 70 min with^3^H-Leucine (Hartmann Analytic, specific activity: 100 Ci mmol^− 1^, final concentration: 20 nmol L^− 1^). Incubations were processed by the centrifugation method and a liquid scintillation counter (Packard Tri-Carb 2900 TR) after termination with trichloroacetic acid at a final concentration of 5%^69^. BBP was estimated using a conversion factor of 1.5 kg C mol leucine^− 1^ assuming no intracellular isotope dissolution^70^.

### Calculations – physical parameters

Temperature and salinity profiles (Fig. 1) were generated by applying a linear fit between monthly measurements in 1-day-steps and a meter-wise fit in 1-m-steps using Matlab^®^ 2023a. Interpolation of the data was performed with monthly sampling intervals. Gap months are shown in white. Stratification was calculated using the temperature gradient ∇T as criterion for stratification (∇T ≥ 0.6 °C m^− 1^ indicates stratification)^27^. Long-term changes in stratification with respect to the season (length, timing etc.) in the Baltic Sea have mainly been attributed to changes in the thermocline due to warming^5^. Hence, here the strength of the thermocline is calculated. Results of the stratification calculation including the length of the stratified season are listed in the supplement (Table S2). All data within this time frame were regarded as data from the stratified season, while data from outside of these time frames were considered data from the mixed season.

To investigate whether very high seawater temperatures could be categorized as heatwaves, a similar approach to Hobday, et al.^28^ was applied. Heatwaves were calculated on the basis of the long-term dataset from 1957 to 2019 with the lower boundary defined by the 0.9 quantile between 1957 and the respective year. The boundaries of the heatwave categories were calculated according to Hobday, et al.^28^ with all measurements falling below the 0.9 quantile or in category I. The results for 1 and 25 m are shown in the supplement (Figure S2). Additionally, the deviation from the mean from 1957 to the respective year of each measured month was calculated (“heatwave indicator”). Deviations above the mean indicate stronger warming than average temperatures, while deviations below the mean indicate cooler months (see Fig. 5).

### Statistical analysis

All statistical analyses were performed using Matlab^®^ 2023a. Before applying statistical methods, data were tested for all requirements of parametric statistics (e.g. linear regression) including normal distribution using the Lillifors test. Data were further normalized before statistical analysis for comparison according to the following equation using the standard deviations and means of each dataset (µ – mean, $\:{\upsigma\:}$ – standard deviation, Eq. 1):

1 $$\:{data}_{norm}=\frac{data-\mu\:}{\sigma\:}$$

A Principal Component Analysis (PCA) was performed on the normalized dataset except for DON and DOC due to their limited data availability (measurements only from 2016 on) to obtain a first estimation for drivers and patterns in the combined dataset. The first two components were mainly driven by salinity (PC1) and temperature (PC2). The PCA revealed that data clustered according to the seasons i.e. mixed vs. stratified. The four parameters salinity, oxygen (PC1), and temperature and BBP (PC2) were tested for differences between the mixed and stratified season by performing a one-way ANOVA on the normalized datasets. The results are shown in the supplementary material.

For further testing relationships between each dataset, the Spearman’s rank correlation coefficient (r_s_) with a significance level of *p* ≤ 0.05 was applied, since the datasets did not meet the necessary requirements for applying linear correlation (i.e. autocorrelation, typical for time-series data). When reported in the study using asterisks, significance levels were listed as follows: * for *p* ≤ 0.05, ** for *p* ≤ 0.01, *** *p* ≤ 0.001, and **** for *p* ≤ 0.0001.

Type II regression linear fits were applied to the DOC and DON data to investigate the production or degradation pathways of DOM at BE^40^ using the gmregress function^71^.

The Matlab^®^ tool fitting box was used to explore best fits for BBP- and oxygen data for the mixed and the stratified seasons, which were exponential fits in both cases (best R^2^). The built-in poly2 and predint functions were used for plotting, to calculate the statistics of the fit, and to further calculate the 95-%-confidence-intervals.

Moving averages (along a 12 months frame) were used to determine long-term trends by removing the seasonality in the bottom layer (25 m) BE data from 1991 to 2019. This time frame was chosen due to the availability of bacterial abundance- and BBP data from Hoppe et al.^26^ determined at the same station in the same depths. While the methods for temperature and oxygen determination in our study and during Hoppe et al.^26^ were the same, bacterial abundance and BBP were determined using different methods. In Hoppe et al.^26^, bacterial abundance was determined using epifluorescence microscopy, while BBP was determined by thymidine incorporation. While epifluorescence microscopy has been shown to be well comparable to flow cytometry^61,62^, thymidine incorporation may overestimate BBP in comparison to leucine incorporation^63^. The slopes of the trends from the two time periods may therefore not be directly comparable but the separate trend estimates can provide estimates for the direction of the trends. The best fits (based on their R^2^) for all calculated moving averages were linear fits. The Mann-Kendall Test^72^ was applied to test for significance in the calculated trends from the moving averages (significance level *p* ≤ 0.05), which is a non-parametric test for monotonic trends. The results are listed in the supplement (Table S4). Trends for bacterial abundance and BBP were calculated separately for the two measurement periods due to the long sampling break. Temperature and oxygen trends were additionally calculated for the same time period for comparison. Furthermore, to determine trends for the stratified season when oxygen reduction is strongest, linear trends were calculated for the months August or September between 1991 and 2008, and 2013 and 2019, and tested using the Mann-Kendall Test (Table S4).

## Electronic supplementary material

Below is the link to the electronic supplementary material.

Supplementary Material 1

## Data Availability

All data are available at PANGAEA with the following DOIs: 10.1594/PANGAEA.855693 (hydrochemistry 1957–2014), https://doi.pangaea.de/10.1594/PANGAEA.973020 (hydrochemistry 2015–2023), and https://doi.pangaea.de/10.1594/PANGAEA.972124 (DOM, BBP, bacterial abundance 2013–2019). Furthermore, data are available in the database of the Boknis Eck time series station (www.bokniseck.de).
