# Supplementary material for: Enhanced warming and bacterial biomass production as key factors for coastal hypoxia in the southwestern Baltic Sea
Source: Sci Rep. 2024 Nov 27;14:29442. doi: 10.1038/s41598-024-80451-w (PMC11603197; doi:10.1038/s41598-024-80451-w)
Supplement: Supplementary file 1 — Supplementary Material 1 [file 41598_2024_80451_MOESM1_ESM.pdf]

SUPPLEMENTARY INFORMATION

Manuscript: "Enhanced warming and bacterial biomass production as key factors for coastal hypoxia in the southwestern Baltic Sea" by Hepach et al.

Baltic Sea map

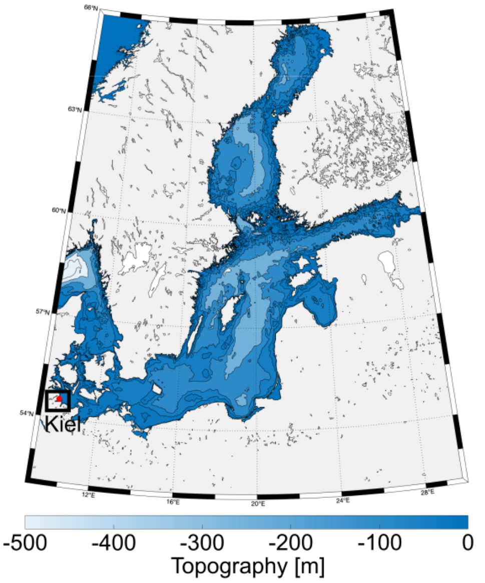

Figure S1. Map of the Baltic Sea including the location of Boknis Eck. The blue scale shows the Baltic Sea topography (see colorbar). Boknis Eck is located in the southwestern Baltic Sea.

Table S1. Means (range) of all measurements, and of the depth profiles (over the standard depths 1, 5, 10, 15, 20 and 25 m) with minimum (min profile) and maximum (max profile) values for each listed parameter between 2013 and 2019 at the BE time series station (mean ± standard deviation over the 6 depths).

Parameter [unit]	Mean total (range)	Mean min profile (month/year)	Mean max profile (month/year)
Temperature [°C]	9.7<br>(0.6 - 21.8)	0.9 ± 0.3<br>(04/2013)	16.8 ± 2.7<br>(09/2016)
Salinity [PSU]	18.9<br>(12.4 - 25.7)	13.4 ± 0.2<br>(04/2018)	24.2 ± 0.6<br>(01/2015)
Oxygen [µmol L^{-1}]	269.2<br>(0.0 - 443.4)	69.8 ± 56.6<br>(09/2017)	417.4 ± 19.4<br>(04/2013)
NO_x [µmol L^{-1}]	2.2<br>(0 - 18.5)	0.1 ± 0.0<br>(08/2014)	0.1 ± 0.0<br>(08/2014)
NH_4^+ [µmol L^{-1}]	1.2	0.0 ± 0.0	7.0 ± 13.4

	(0 - 40.4)	(06/2014)	(10/2014)
PO_4^{3-} [µmol L^{-1}]	0.7<br>(0 - 11.3)	0.1 ± 0.0<br>(04/2018)	2.2 ± 3.8<br>(10/2014)
Bacterial cell counts [cells mL^{-1}]	1.2 x 10^6<br>(0.2 x 10^6 - 4.8 x 10^6)	0.3 x 10^6 ± 0.1 x 10^6<br>(01/2015)	2.7 x 10^6 ± 1.8 x 10^6<br>(04/2016)
Bacterial biomass production [mg C m^{-3} d^{-1}]	1.9<br>(0.2 - 11.7)	0.3 ± 0.0<br>(01/2015)	5.6 ± 3.1<br>(08/2016)
DOC [µmol L^{-1}]	256.9<br>(198.9 - 430.5)	215.0 ± 7.5<br>(03/2019)	354.5 ± 12.9<br>(03/2019)
DON [µmol L^{-1}]	17.1<br>(10.0 - 31.5)	12.1 ± 0.2<br>(10/2019)	20.6 ± 1.5<br>(01/2018)
DOC:DON	15.4<br>(7.5 – 22.4)	11.9 ± 0.9<br>(01/2018)	20.6 ± 1.2<br>(10/2019)

<u>Stratification</u>

Table S2. Onset of stratification (and the depth, at which the stratification is strongest) and the last month of the stratified season in each year, along with the stratification indicator ∇T with a threshold value of ≥ 0.6 °C m^{-1} at the start and the end of the season.

**Year**	**Month of onset stratification (lower layer)**	**Last month of stratification**	**∇T [°C m^{-1}]**
2013	June (20 m)	September	1.5
2014	June (20 m)	November	0.7
2015	May (20 m)	November	0.7
2016	June (10 m)	October	1.0
2017	June (20 m)	October	0.8
2018	June (10 m)	October	1.3
2019	June (20 m)	October	0.7

Heatwaves

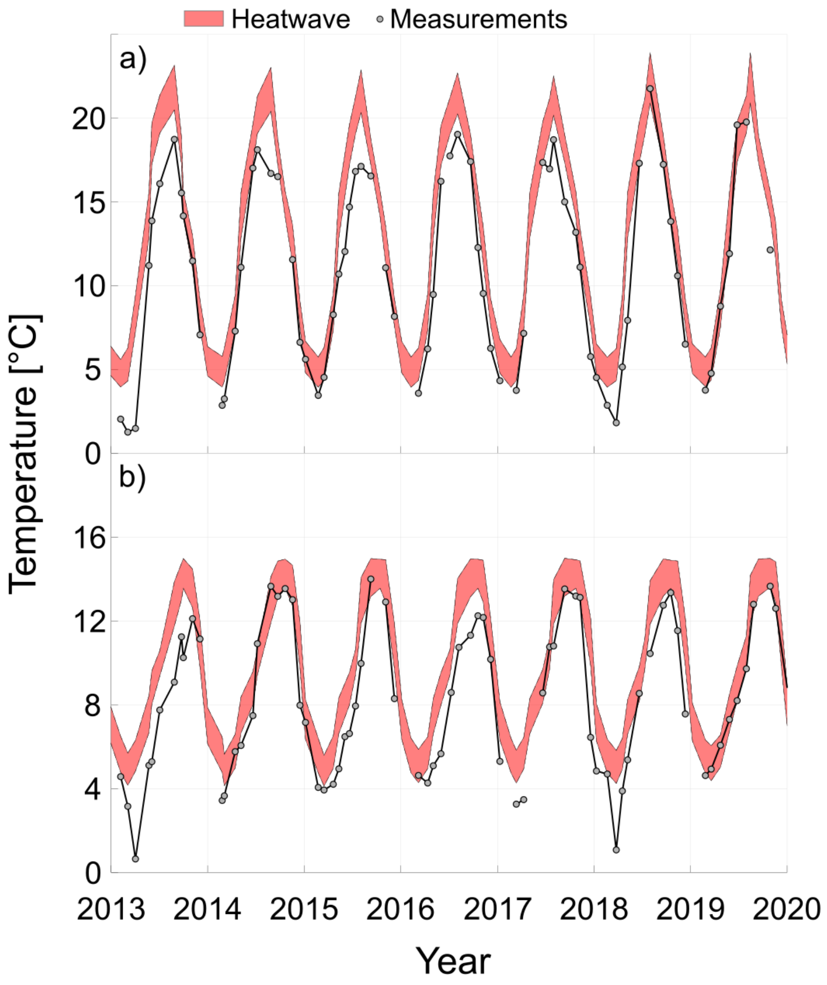

Figure S2. Temperature measurements in 1 m (a) and 25 m (b) along with the range for category I marine heatwaves based on the long-term measurements from 1957 until 2019.

Distribution of DOC, TDN and DON

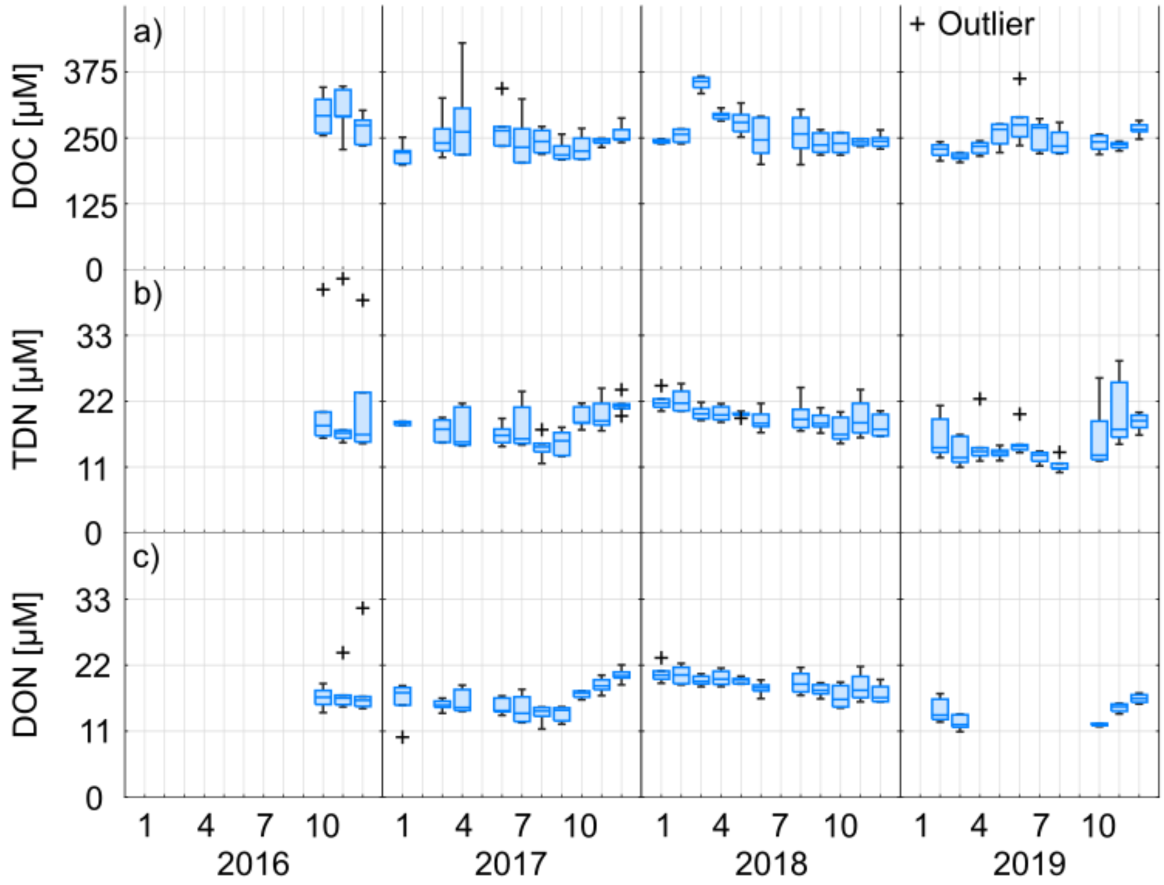

Figure S3. DOC in a), TDN in b, and TDN corrected with DIN to DON in c) with concentrations from the boxplots on the left-hand side and integrated concentrations on the right-hand side (dots). Crosses indicate outliers.

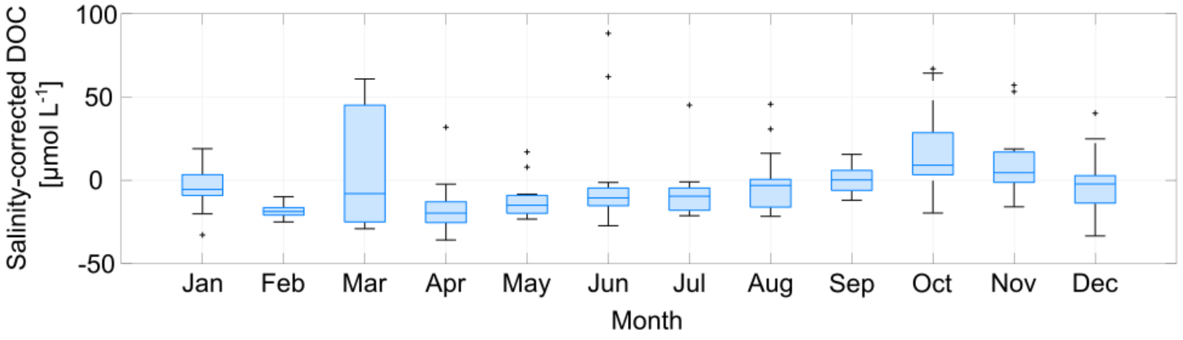

Figure S4. Plot of theoretical autochthonous DOC input.

The theoretical autochthonous DOC input (salinity-corrected DOC) was calculated using the relationship of DOC and salinity. The positive difference between the measured DOC and the theoretical DOC derived from the linear equation in Fig. 3a (everything above 0) was regarded as the additional input from autochthonous sources. The correction was calculated for all DOC

values between 2016 and 2019 from all depth, and values included in the boxes are summarized for each month. A similar approach was used by Osterholz et al.[1].

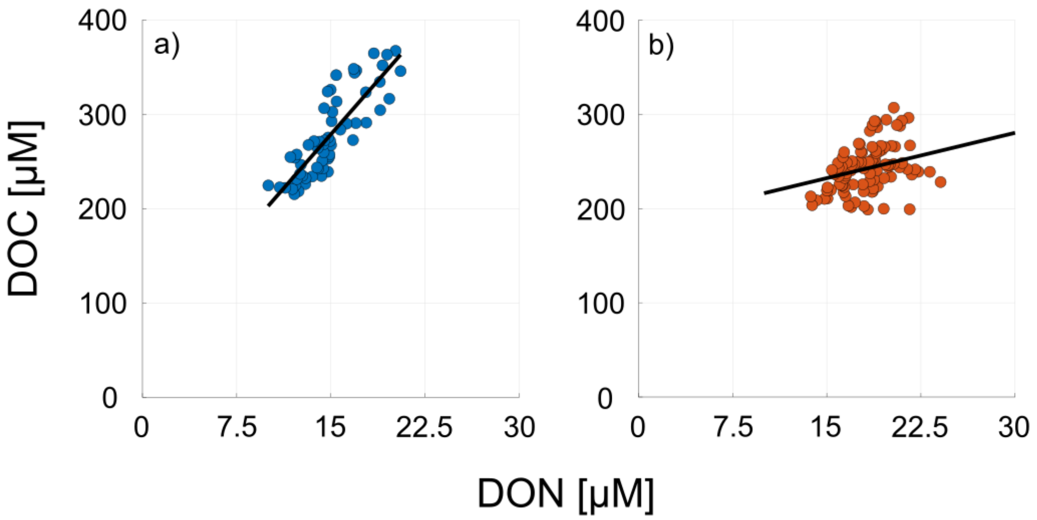

Figure S5. DOC vs DON for DOM above the North-Sea-line (99 data points) in a) and below the North-Sea-line (61 data points) in b). The regression lines were calculated from model type II regressions according to Hopkinson and Vallino, 2005[2].

Drivers of BBP

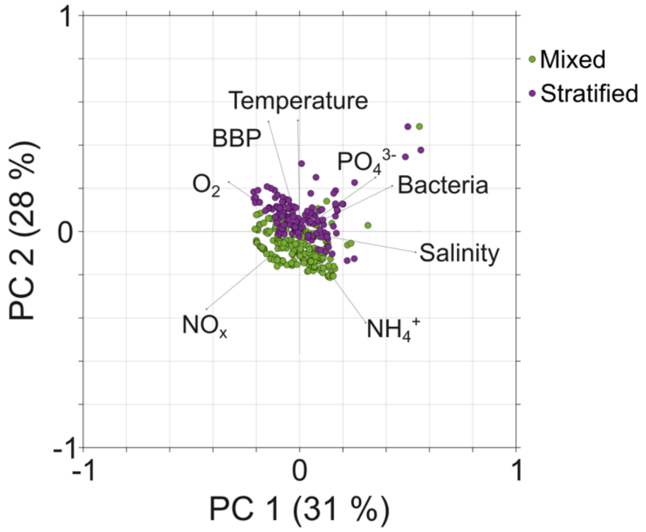

Figure S6. PCA of the whole data set. Colors show seasonal groups.

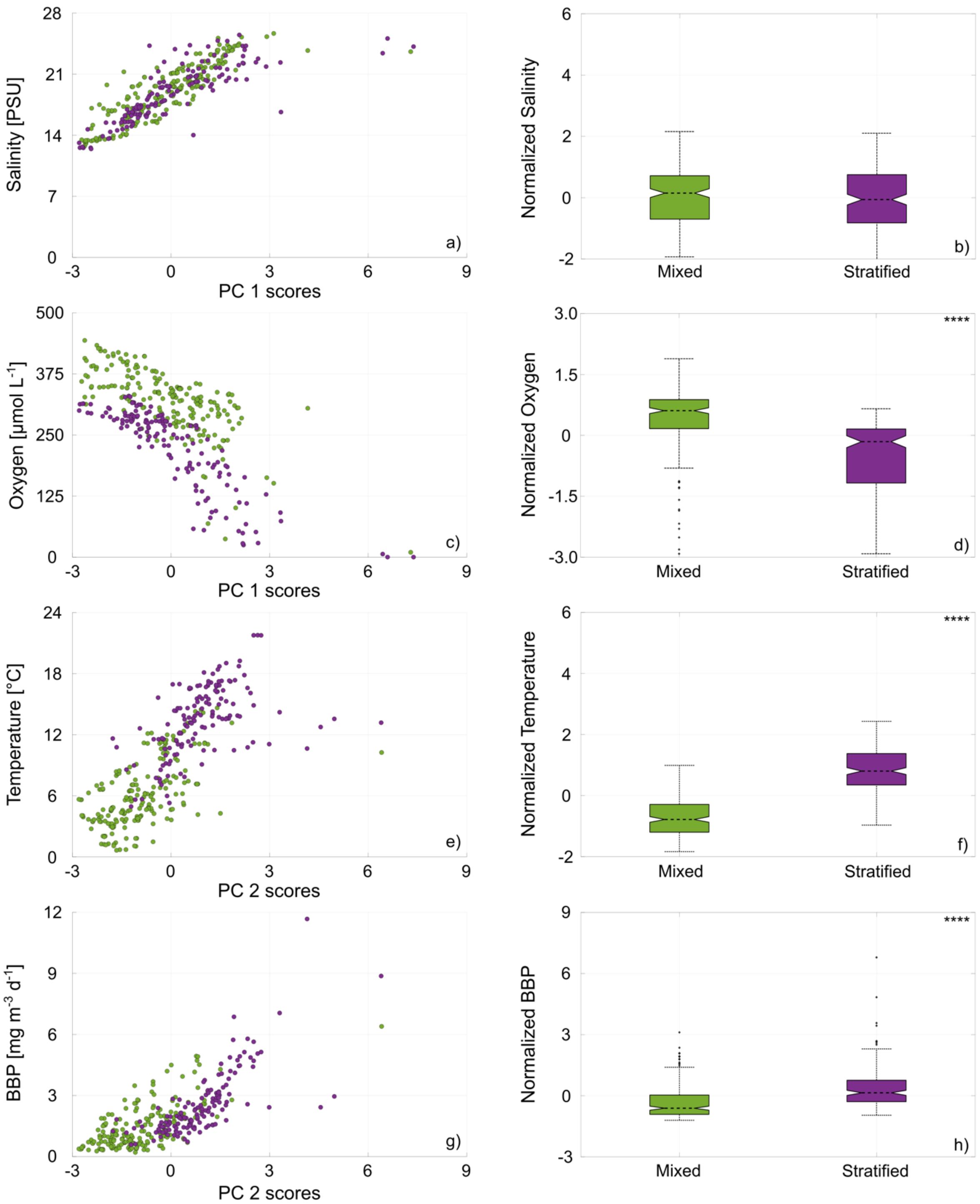

Figure S7. Salinity (a, b), oxygen (c, d), temperature (e, d) and bacterial biomass production (g, h) vs component 1 from the PCA (salinity, oxygen) and component 2 (temperature, bacterial biomass production) along with box plots for the mixed and stratified seasons. The asterisks indicate the significance level between the seasons for the one-way ANOVA.

The plot of the Principal Component Analysis (PCA) revealed a potential optical clustering of the data set into the mixed and the stratified season. Correlation analysis of the data and the

calculated components showed that component 1 (PC1) was mainly driven by salinity (r_s = 0.82, p ≤ 0.05, Fig. S4a), followed by oxygen (r_s = -0.69, p ≤ 0.05, Fig. S4c). Temperature (r_s = 0.75, p ≤ 0.05, Fig. S4e) and bacterial biomass production (r_s = 0.75, p ≤ 0.05, Fig. S4g) are the most significant drivers of component 2 (PC2). A one-way ANOVA was performed to test whether there were significant differences between the two seasons (mixed and stratified) with respect to the tested parameter. The tested parameters were: salinity, oxygen, temperature and BBP. The one-way ANOVA showed significant differences (p < 0.0001) between the two groups (mixed and stratified) for oxygen, temperature and BBP. Salinity showed no season-dependent differences.

Bacterial abundance data showed the most significant relationships to component 3 (r_s = 0.60, p ≤ 0.05) and PC1 (r_s = -0.50, p ≤ 0.05), indicating that the drivers of bacterial biomass production are not solely related to bacterial abundance (bacterial biomass production and PC1: r_s = -0.22, p ≤ 0.05; bacterial biomass production and component 3: r_s = 0.19, p ≤ 0.05).

Table S3. Fit parameter for Fig. 5a-d with $y = a \cdot e^{b \cdot x}$. Furthermore, the coefficient of determination per fit is given (r^2). The Spearman's rank coefficient (r_s) including the significance level (p) of the relationship between the measured data and the modeled data from the fit are provided in the last column. The number of data points in a) and b) is 37, while the number of data points in c) and d) is 26.

Panel number	a	b	r^2	r_s, p
a)	0.38	0.16	0.13	-0.09, 0.60
b)	298	-0.16	0.23	0.30, 0.07
c)	0.06	0.26	0.36	0.69***
d)	323	-0.65	0.70	0.84****

<u>Long term fits and trends</u>

Table S4. P-values from the Mann-Kendall Tests (significance level 0.05) for the long-term trends fitted to the 12-months moving averages, along with the rates (slope) of increase (+) or reduction (-) per year for each parameter (temperature and oxygen including all data between 1991 and 2019, for comparison for the periods 1991 – 2008 and 2013 – 2019 (omitting the years 2009 – 2012), bacterial abundance and bacterial biomass production for the time periods 1991 – 2008 and 2013 – 2019). Furthermore, trends and Mann-Kendall Tests for only data from August (temperature, bacteria, BBP) and September (oxygen) are listed.

Parameter	Mann-Kendall Test	Rate yr^{-1}
Temperature, all data	$p = 0$	+ 0.05 °C
Temperature, 1991 - 2008	$p = 0$	+ 0.07 °C
Temperature, 2013 - 2019	$p \leq 0.0001$	+ 0.23 °C
Oxygen, all data	$p = 0.01$	- 0.14 µmol L^{-1}
Oxygen, 1991 - 2008	$p \leq 0.0001$	- 1.90 µmol L^{-1}
Oxygen, 2013 - 2019	$p = 0.01$	- 2.15 µmol L^{-1}
Bacterial abundance, 1991 - 2008	$p = 0.005$	- 1.0 x 10^4 cells mL^{-1}
Bacterial abundance, 2013 - 2019	$p \leq 0.0001$	+ 8.4 x 10^4 cells mL^{-1}
BBP, 1991 - 2008	$p \leq 0.0001$	+ 0.06 mg C m^{-3} d^{-1}
BBP, 2013 - 2019	$p = 0.73$	+ 0.01 mg C m^{-3} d^{-1}
Temperature 08/1991 – 09/2008	no p-value	+ 0.04 °C
Temperature 08/2013 – 09/2019	$p = 0.15$	+ 0.26 °C
Oxygen 08/1991 – 09/2008	$p = 0.14$	- 0.40 µmol L^{-1}
Oxygen 09/2013 – 09/2019	$p = 0.01$	- 3.36 µmol L^{-1}
Bacterial abundance 08/1991 – 09/2008	$p = 0.75$	- 7.1 x 10^4 cells mL^{-1}
Bacterial abundance 08/2013 – 09/2019	$p = 0.11$	+ 2.4 x 10^5 cells mL^{-1}
BBP 08/1991 – 09/2008	$p = 1$	+ 0.04 mg C m^{-3} d^{-1}
BBP 08/2013 – 09/2019	$p = 0.01$	+ 0.80 mg C m^{-3} d^{-1}

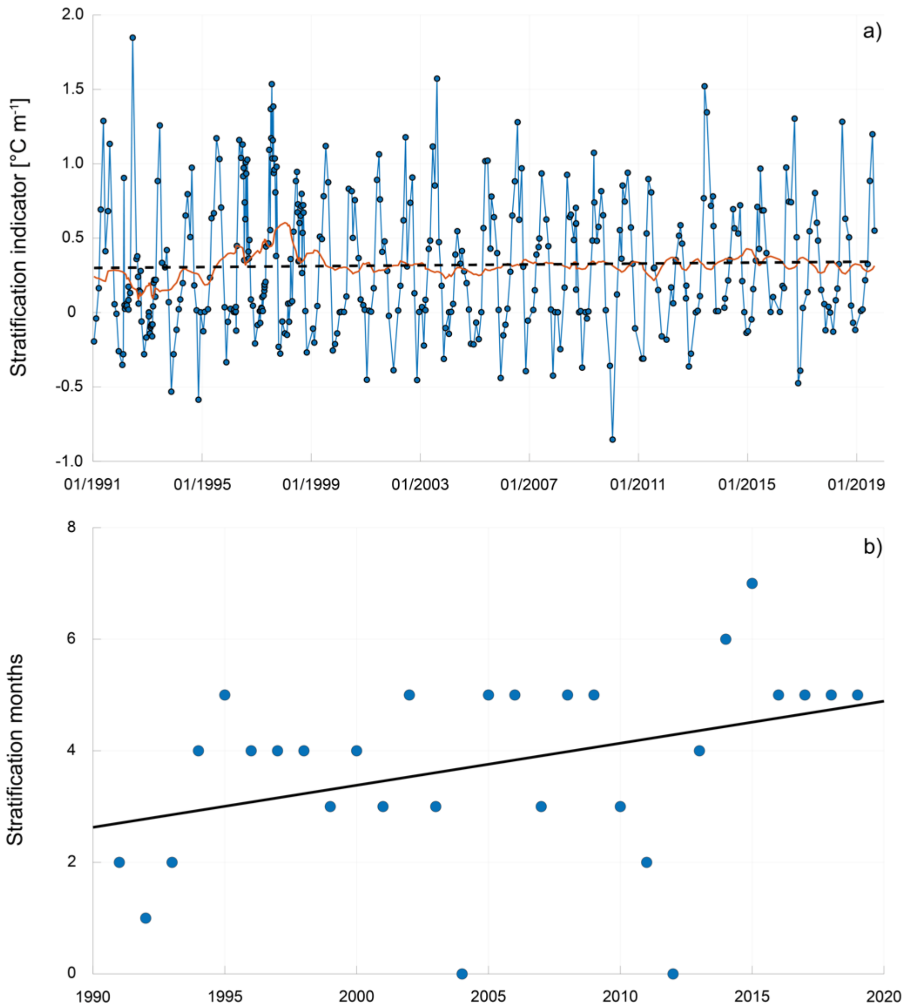

Figure S8. The stratification indicator from 1991 to 2019 in a) with the orange-red line as the 3-year average and the dotted line as the trend (positive). The number of months, in which the water column is stratified is shown in b) with the black line as the trend line.

References

1 Osterholz, H. *et al.* Nearshore Dissolved and Particulate Organic Matter Dynamics in the Southwestern Baltic Sea: Environmental Drivers and Time Series Analysis (2010–2020). *Frontiers in Marine Science* **8**, doi:10.3389/fmars.2021.795028 (2021).

2 Hopkinson, C. S. & Vallino, J. J. Efficient export of carbon to the deep ocean through dissolved organic matter. *Nature* **433**, 142-145, doi:10.1038/nature03191 (2005).
